# Vaccination coverage rates of military personnel worldwide: a systematic review of the literature

**DOI:** 10.1007/s00420-020-01559-w

**Published:** 2020-06-19

**Authors:** Jana Nele Arnold, Nils Gundlach, Irina Böckelmann, Stefan Sammito

**Affiliations:** 1 Bundeswehr Hospital Hamburg, Hamburg, Germany; 2 Occupational Medicine, Otto-Von-Guericke University Magdeburg, Magdeburg, Germany; 3 Medical Clinic Rotenburg, Rotenburg, Germany; 4 Air Force Centre of Aerospace Medicine, Department I 3, Flughafenstraße 1, 51147 Cologne, Germany

**Keywords:** Bundeswehr, Prevention, Vaccine, Military personal

## Abstract

**Objectives:**

Due to the professionally specific risk of infection in the armed forces, recommendations for vaccination are usually adapted for soldiers and are subject to special regulations. Little data is available on scientifically measured vaccination coverage of soldiers.

**Methods:**

A systematic literature research was carried out in the PubMed database using the search terms “army” or “military” or “Bundeswehr” and “vaccination” or “vaccine”. Studies covering the period from 1990 to 2018 that contain statements on vaccination coverage rates of soldiers were identified. Twenty-two out of the initially found 1801 results were used.

**Results:**

The studies found were conducted in nine different countries with eight out of the 22 studies originating from the USA. The size of study was between 180 and 32,502 subjects. On average, the vaccination rates determined in the studies were between 26.8 and 94.7%. Hepatitis A coverage was lowest (a minimum of 11.3%) and tetanus vaccination coverage was highest (with a maximum of 94.7%). Vaccination rates decreased with increasing age and coverage tended to be lower for men than for women. The term of service did not have a significant effect on vaccination rates.

**Conclusions:**

On the whole, most studies referred to recruits. They showed high vaccination rates for standard vaccinations and lower vaccination rates for indication and seasonal vaccinations. However, there were also vaccination gaps of temporary-career volunteers. This leads to a considerable effort at the armed forces to complete vaccine protection in case of a short-term operational commitment.

## Introduction

Vaccinations are some of the most effective preventive measures with few side effects to prevent infectious diseases. The effectiveness of vaccinations for the population depends on high vaccination coverage.^1^ A vaccination rate of 75–94% is necessary to achieve a so-called herd immunity (Fine et al. 2011). This ensures that even people who cannot be vaccinated for medical reasons can receive the best possible protection against diseases. Vaccination has drastically reduced the global number of cases of many infectious diseases. WHO data, for example, show a reduction in the documented pertussis cases from 1,982,355 in 1980 to 143,963 in 2017. Only 96 cases of polio were reported in 2017, compared to 52,795 cases in 1980 (World Health Organization 2018). Furthermore, the WHO records the expected annual vaccination rates for different diseases. These data are officially submitted by the member states every year. The global vaccination rates are between 28% for rotavirus and 90% for the first tetanus vaccine (World Health Organization 2018). Data on the civilian population in Germany show a considerable lack of vaccine protection. The DEGS1-Study from 2013 reveals a vaccination from 71.4% (69.8–72.9%) against tetanus within the last 10 years and from 57.1% (53.3–58.9%) against diphtheria within the last 10 years (Poethko-Müller and Schmitz 2013). This lack of vaccine protection is repeatedly seen in outbreaks of vaccine-preventable diseases such as the outbreak of measles in Cologne, Germany between January and August 2018 with 139 cases of measles (Osagie-Paech et al. 2019).

For soldiers^2^ there are additional occupational risks of infection which can be prevented by vaccinations (Bundeswehr Medical Service Headquarter 2014; German Ministry of Defense 2014). It is plausible to assume that existing vaccination rates are generally above the rates of the general population due to the special employment of soldiers and the requirement of rapid deployability. However, there are also outbreaks of vaccine-preventable diseases among soldiers. From 2010 to 2011, there was a large outbreak of measles in the French military with the incidence increasing from 1:100,000 to 10.1:100,000 in 2010 and 41.4:100,000 in 2011 (Mayet et al. 2013). Furthermore, current results from the German armed forces (Bundeswehr) show that there are also vaccination deficiencies in this occupational group (Arnold et al. 2017). The following sections of this systematic review will examine the scope of vaccine requirements for soldiers of international armed forces according to published studies.

## Methods

A systematic literature research was carried out to clarify the following two issues:

- What are the vaccination rates of soldiers from other nations? The vaccinations of the so-called basic vaccine protection for Bundeswehr soldiers were used as examples for comparison because the vaccinations listed there are significant in the field of preventive medicine for a large part of the European continent and to facilitate the transferability to regular vaccinations for German soldiers.
- Is there a difference between new employees (recruits) and temporary-career volunteers?

### Inclusion and exclusion criteria

The systematic literature research in the PubMed database was carried out on 20 April 2018 using the search terms “army” or “military” or “Bundeswehr” and “vaccination” or “vaccine” or “Impfung”. German and English articles published after 1 January 1990 were included. Consideration was given to cross-sectional studies dealing with vaccination rates of recruits or temporary-career volunteers. No distinction was made whether the vaccination rates were recorded using questionnaires or serological examination. All studies that did not contain information on vaccinations of the basic vaccine protection of the Bundeswehr [tetanus, diphtheria, poliomyelitis, pertussis, hepatitis A, hepatitis B, measles, mumps, rubella (MMR) tick-borne encephalitis (TBE) and influenza] (Bundeswehr Medical Service Headquarter 2014) were excluded. In addition, the factors that were examined for a statistically significant influence on vaccination rates were documented.

### Systematic literature research

**Fig. 1:**
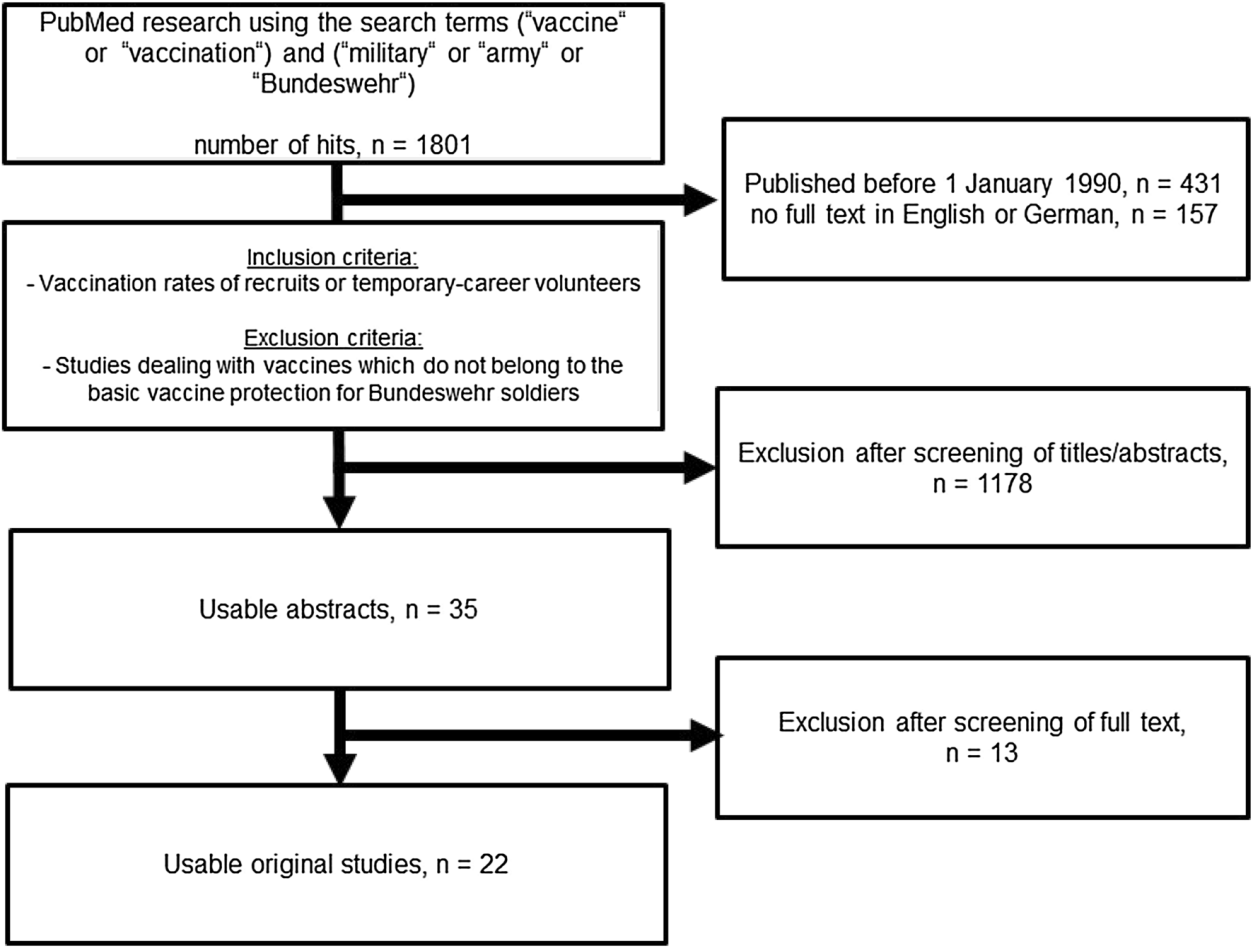
Flowchart on study selection

The systematic search found 1,801 results for the above-mentioned search terms (see Fig. 1). 431 of the articles found were published before 1990 and 157 articles were not available in English or German. After the titles and abstracts of the remaining 1213 results were screened, 35 results were suitable for the issue. Of these, eleven studies were excluded because they deviated from the issue (do not focus on vaccination rates of recruits or temporary-career volunteers or dealing with vaccines which do not belong to the basic vaccine protection for Bundeswehr soldiers). Two studies were excluded because there were no access rights to the full texts. Altogether 22 articles were taken into account in the systematic review.

## Results

Of the 22 studies identified (see Table 1), eight studies come from the US Armed Forces (Clardy 1993; Eick et al. 2008; Kelley et al. 1991; Lewis 2015; Nevin and Niebuhr 2007; Scott et al. 2005; Smoak et al. 1994; Struewing et al. 1993), six studies from the Israel Defence Forces (Arav-Boger et al. 2000; Balicer et al. 2007; Huerta et al. 2006; Levine et al. 2011, 2012, 2015), two studies from the Norwegian Armed Forces (Flugsrud et al. 1997; Vainio et al. 2007) as well as one study from Brazil, Iran, Italy, Saudi Arabia, Spain and Thailand, respectively (Al-Khashan et al. 2011; Arteaga et al. 2010; Gonwong et al. 2016; Hosseini Shokouh et al. 2017; Passos et al. 2011; Rappuoli et al. 1993). With one exception, all studies considered are cross-section surveys of vaccination rates. In deviation from this, Eick et al. (2008) conducted a cohort study from 2000 to 2004. The vaccination rates presented in this review originate from the basic data collection at the beginning of the study.

**Table 1:** Overview of the search results from the systematic literature research

Study	Year	Number of subjects	Country/service	Time at military	Type of data collection	Age	♂	♀
Al-Khashan et al.	2011	2286	Saudi Arabia/all armed services	14.9 ± 7.8 years	Questionnaire	36.3 ± 7.5 years	N/A	N/A
Arav-Boger et al.	2000	533	Israel	Recruits	Seroprevalence	17 or 18 years	59.7%	40.3%
Arteaga et al.	2010	226	Spain	Recruits	Seroprevalence, Questionnaire	20.2 ± 1.7 years	92.5%	7.5%
Balicer et al.	2007	942	Israel/Air Force	N/A	Questionnaire	22.5 ± 5.1 years	80.2%	19.8%
Clardy F	1993	276	USA/Air Force	Recruits	Seroprevalence, Questionnaire	52.5% < 20 years, 41.3% 20–24 years, 6.2% > 24 years	85.5%	14.5%
Eick et al.	2008	3000	USA/All Services	Recruits	Cohort Study, Seroprevalence	49.0% 17–19 years, 39.9% 20–24 years, 8.8% 25–29 years, 2.2% 30–35 years	79.0%	21.0
Flugsrud et al.	1997	1188 soldiers, 695 civilians	Norway	Recruits	Seroprevalence	Age 18–28 years, Average 20.7 years	98.7%	1.3
Gonwong et al.	2016	7760	Thailand	Recruits	Seroprevalence	15.2% 18–20 years, 69.8% 21 years, 13.1% 22–24 years, 1.9% 25–30 years	100%	0%
Hosseini Shokouh et al.	2017	180 military staff, 83 civilians	Iran	N/A	Seroprevalence	27.2% 18–34 years, 32.8% 35–50 years, 40% > 50 years	95.6%	4.4%
Huerta et al.	2006	353	Israel	Recruits	Seroprevalence	95% 18–19 years	56.4%	43.6%
Kelley et al.	1991	1547	USA	Recruits	Seroprevalence, Questionnaire	Alter 17–35 years, 77% < 20 years	73.7%	26.3%
Levine et al.	2011	441	Israel	Recruits	Seroprevalence	95% 18–19 years	57.1%	42.9%
Levine et al.	2012	416	Israel	Recruits	Seroprevalence	95% 18–19 years	57.5%	42.5%
Levine et al.	2015	439	Israel	Recruits	Seroprevalence	95% 18–19 years	56.50%	43.5%
Lewis et al.	2015	32,502	USA/Air Force	Recruits	Seroprevalence	47.0% 17–19 years, 42.9% 20–24 years, 8.3% 25–29 years, 1.3% 30–35 years, 1.3% > 35 years	78.6%	21.4%
Nevin and Niebuhr	2007	2026	USA	Recruits	Seroprevalence	14.1% 18–19 years, 9.3% 20–24 years, 14.0% 25–29 years, 11.8% 30–34 years	82.6%	17.4%
Passos et al.	2011	371	Brazil/Air Force	Recruits	Seroprevalence, Questionnaire, Vaccination card	19–20 years	100.0%	0.0%
Rappuoli et al.	1993	334	Italy	Recruits	Seroprevalence	17–22 years	N/A	N/A
Scott et al.	2005	2400	USA / 55% army, 26% navy, 19% marine Corps	Recruits	Seroprevalence	50% 18–19 years, 25% 20–23 years, 25% 24–35 years,	81.5%	18.5%
Smoak et al.	1994	1961	USA/army	Recruits	Seroprevalence	Average 19.8 years (1989)	59.0%	41.0%
Struewing et al.	1993	1533	USA/navy, Marine Corps	Recruits	Questionnaire	Average 20.3 years (1990)	56.0%	44.0%
Vainio et al.	2007	1405	Norway	Recruits	Seroprevalence	74.1% 17–19 years, 21.4% 20–24 years, 3.7% over 25 years	90.8%	9.2%

*N/A* not available/not applicable

The number of subjects was between 180 in the study by Hosseini Shokouh et al. (2017) and 32,502 in the study by Lewis (2015).

15 studies recorded the vaccination rates via seroprevalence (Arav-Boger et al. 2000; Eick et al. 2008; Flugsrud et al. 1997; Gonwong et al. 2016; Hosseini Shokouh et al. 2017; Huerta et al. 2006; Levine et al. 2011, 2012, 2015; Lewis 2015; Nevin and Niebuhr 2007; Rappuoli et al. 1993; Scott et al. 2005; Smoak et al. 1994; Vainio et al. 2007), three studies collected the data by means of questionnaires (Al-Khashan et al. 2011; Balicer et al. 2007; Struewing et al. 1993), and four studies combined the two procedures (Arteaga et al. 2010; Clardy 1993; Kelley et al. 1991; Passos et al. 2011) for determining the vaccination rates. The study by Arteaga et al. (2010) showed a positive predictive value of 98.8% for tetanus for the existence of seropositivity when stating a previous vaccination in the questionnaire.

The majority of the articles did not contain information on the armed services of the subjects, only seven studies provided information on this. The studies by Balicer et al. (2007), Clardy (1993), Lewis (2015) and Passos et al. (2011) examined Air Force members, the study by Smoak et al. (1994) Army soldiers and the study by Struewing et al. (1993) Navy/Marine Corps soldiers. The study by Scott et al. (2005) dealt with the armed service of each participant and conducted an additional sub-group analysis with regard to the vaccination status. This showed no significant differences in vaccination rates between the individual armed services.

Most studies were conducted with recruits. Only the three studies by Al-Khashan et al. (2011), Balicer et al. (2007) and Hosseini Shokouh et al. (2017) examined soldiers who had been serving in the armed forces for quite some time. In this context, it must be stressed that the study by Al-Khashan et al. (2011) was the only one that considered the period of service at the time of study which was 14.9 ± 7.8 years.

The breakdown of study participants by age was carried out differently in the studies included. Inconsistent age intervals make the comparability of the studies more difficult. Altogether, only six studies described age groups over the age of 30 (Al-Khashan et al. 2011; Eick et al. 2008; Hosseini Shokouh et al. 2017; Kelley et al. 1991; Lewis 2015; Nevin and Niebuhr 2007). The examinations which captured averages showed values between 19.9 and 36.3 years (Arteaga et al. 2010; Balicer et al. 2007; Flugsrud et al. 1997; Smoak et al. 1994; Struewing et al. 1993). The study population mainly consisted of men with a percentage between 56 and 100%.

The vaccination rates presented in the studies (see Table 2) were between 10.4% and 94.7%. The vaccination rates for tetanus (94.0–94.7%), diphtheria (77.0–77.4%), poliomyelitis (85.4%), mumps (80.3–91.6%), measles (78.5–92.3%) and rubella (81.6–94.8%) were very high. The vaccination rates for pertussis (58.6%) and hepatitis B (31.5–84.0%) were in the mid-range. With 78.3% or 84.0%, the rates for hepatitis B were significantly higher in the studies by Arteaga et al. (2010) and Passos et al. (2011) than those in the study by Scott et al. (2005) with 31.5%. In contrast, the vaccination rates for influenza (17.8–48.5%) and hepatitis A (10.4–12.0%) were low. The weighted averages were then calculated in relation to the relevant number of subjects *n*. These were between 11.9% for hepatitis A and 94.4% for tetanus.

**Table 2:** Vaccination rates for soldiers from different armed forces as a percentage and the resulting weighted averages for vaccinations which is the total of the vaccination rate multiplied by the quotient resulting from the number n and the number of all subjects where the vaccination rate for this disease was recorded

Study	Number n	Tetanus	Diphtheria	Polio	Pertussis	HepA	HepB	Mumps	Measles	Rubella	Influenza
Al-Khashan et al. (2011)	2286										17.8%
Arav-Boger et al. (2000)	533				58.6%						
Arteaga et al. (2010)	226	94.2%	77.4%			10.4%	78.3%				
Balicer et al. (2007)	942										48.5%
Clardy (1993)	276							83.0%	80.4%	85.5%	
Eick et al. (2008)	3000							91.6%	86.1%	94.8%	
Flugsrud et al. (1997)	1188								92.3%		
Gonwong et al. (2016)	7760								78.5%		
Hosseini Shokouh et al. (2017)	180	94.0%	77.0%								
Huerta et al. (2006)	353							83.3%			
Kelley et al. (1991)	1547			85.4%				84.4%	79.3%	82.5%	
Levine et al. (2011)	441							83.7%			
Levine et al. (2012)	416									87.7%	
Levine et al. (2015)	439								85.7%		
Lewis et al. (2015)	32,502							80.3%	81.6%	82.1%	
Nevin and Niebuhr (2007)	2026					12.0%					
Passos et al. (2011)	371						84.0%				
Rappuoli et al. (1993)	334	94.7%	77.1%								
Scott et al. (2005)	2400						31.5%				
Smoak et al. (1994)	969 (1989)							83.8%	78.7%	81.6%	
	992 (1990)							85.3%	87.5%	85.7%	
Struewing et al. (1993)	1533							87.7%	82.2%		
Vainio et al. (2007)	1405								89.3%		
Weighted average		94.4%	77.2%	85.4%	58.6%	11.9%	41.5%	81.8%	81.9%	83.2%	26.8%

In the studies examined, several reasons were given for the lack of vaccination. In the study by Al-Khashan et al. (2011) 50.3% of the interviewed persons did not know whether there was current vaccine protection against influenza. In the study by Balicer et al. (2007) 10.8% of the subjects who had refused vaccination were asked for their reasons. 37.7% were afraid of adverse effects of vaccination, 32.1% did not believe in the effectiveness of vaccination, 23.1% rejected vaccination generally and 5.1% stated other reasons.

Most studies examined statistically significant deviations of vaccination rates taking into account different influencing factors (Table 3). The variables differed considerably from study to study; age and gender of the subjects were most frequently and most consistently analyzed.

**Table 3:** Breakdown of the potential influencing factors age and gender into significant or insignificant effects on seropositivity

Study	Significant variables	Insignificant variables
Al-Khashan et al. (2011)		Age (*p* = 0.849)
Arav-Boger et al. (2000)	Women > men (*p* = 0.002)	
Arteaga et al. (2010)		Age, gender
Balicer et al. (2007)	Age (age ↑ → seropositivity ↓) (*p* = 0.001)	Gender (*p* = 0.872)
Clardy (1993)	Age (age ↑ → seropositivity ↑), women > men	
Eick et al. (2008)	Age (age ↑ → seropositivity ↓) (*p* ≤ 0.05)	Gender
Flugsrud et al. (1997)		Age (*p* > 0.05)
Gonwong et al. (2016)	Age (age ↑ → seropositivity ↓) (*p* < 0.05)	
Hosseini Shokouh et al. (2017)	Age (age ↑ → seropositivity ↓) for tetanus (*p* < 0.01), for diphtheria (*p* = 0.047), for diphtheria men > women (*p* = 0.049)	Gender in case of tetanus
Huerta et al. (2006)		Gender (*p* = 0.13)
Kelley et al. (1991)	Age (age ↑ → seropositivity ↓) (*p* = 0.005), Women > Men	
Levine et al. (2011)		Gender (*p* = 0.31), age (*p* = 0.18)
Levine et al. (2012)	Women > men (*p* < 0.01)	
Levine et al. (2015)		Gender
Lewis et al. (2015)	Measles (age ↑ → seropositivity ↓) (*p* < 0.001), Mumps and Rubella (age ↑ → seropositivity ↑) (*p* = 0.002), women > men (*p* < 0.001)	
Nevin and Niebuhr (2007)	Age (*p* = 0.015)	Gender
Passos et al. (2011)	Age (*p* = 0.035)	
Rappuoli et al. (1993)		
Scott et al. (2005)	Age (age ↑ → seropositivity ↓) (*p* > 0.01), women > men (*p* > 0.01)	
Smoak et al. (1994)	Age (age ↑ → seropositivity ↓), women > men	
Struewing et al. (1993)	Measles/Mumps in women (age ↑ → seropositivity ↓) (*p* < 0.05)	Measles/Mumps in men
Vainio et al. (2007)		Gender

If available in the studies, the relevant probability coefficient (*p*) is given

With regard to age, seven studies revealed a significantly (*p* < 0.05) lower seropositivity (Balicer et al. 2007; Eick et al. 2008; Gonwong et al. 2016; Hosseini Shokouh et al. 2017; Scott et al. 2005; Smoak et al. 1994; Struewing et al. 1993) in older age groups. The study by Clardy (1993) showed a higher seropositivity among older age groups and the study by Lewis (2015) returned contradictory results for mumps, rubella and measles. Four other studies revealed no significant effect of age (Al-Khashan et al. 2011; Arteaga et al. 2010; Flugsrud et al. 1997; Levine et al. 2011).

In eight studies, gender proved to be a significant influencing factor. In seven studies, seropositivity was higher in women than in men (Arav-Boger et al. 2000; Clardy 1993; Kelley et al. 1991; Levine et al. 2012; Lewis 2015; Scott et al. 2005; Smoak et al. 1994). Only in the study by Hosseini Shokouh et al. (2017) men were better immunized against diphtheria than women. Nine studies showed no statistically significant effect of gender (Arteaga et al. 2010; Balicer et al. 2007; Eick et al. 2008; Hosseini Shokouh et al. 2017; Huerta et al. 2006; Levine et al. 2011, 2015; Nevin and Niebuhr 2007; Vainio et al. 2007).

Other variables in the different studies were, for example, rank, armed service, education, income, country/region of origin, skin color or ethnic background. These could not be considered in the analysis due to their inconsistency.

## Discussion

This systematic review provides the first overview of scientific studies which dealt with the vaccination rates of active-duty military personnel. On the whole, the studies show that there is still a need for improvement in the armed forces with regard to the completion of vaccination rates of soldiers, particularly since they are exposed to significant risks due to their professional activities.

Information on the current vaccination recommendations from the different countries were only available for the civilian population. There was no access to the military provisions because, like the recommendations of the Bundeswehr, they are published in non-accessible guidelines and are partially treated as classified information.

Most studies which could be identified in the context of this systematic literature research examined vaccination rates of recruits. This probably results from the fact that recruits are a cohort which can be easily analyzed in the context of their pre-employment medical examination. There is little data available on temporary-career volunteers.

In the studies found, both information from the soldiers and serological results were used to determine the vaccine protection of the subjects. A low serological titer does not necessarily mean that no vaccination has been carried out but several studies show a high degree of conformity between anamnestic information and serology with positive predictive values over 90% (Alp et al. 2012; Ferson et al. 1994). For this reason, both methods were equally used in this review.

Altogether, most variable results were obtained with regard to vaccination rates of soldiers. Vaccination coverage rates for the well-established vaccines of tetanus, diphtheria, poliomyelitis, mumps, measles and rubella were mostly very high. In this case, herd immunity can be assumed. Apart from the long time availability of the vaccine, this could be because these vaccines are administered systematically from early childhood and thus high seropositivity can be assumed in young adults. Furthermore, these are vaccinations that show long phases of vaccine protection after basic immunization has been completed.

Particularly noticeable were the low vaccination rates for hepatitis A and influenza. This can be explained by the fact that influenza is a seasonal vaccine which needs to be refreshed annually (Impfkommission 2017). Hepatitis A, however, is a vaccine which is not one of the standard vaccines in childhood in the surveyed countries (Spain and USA) (Centers for Disease Control and Prevention 2018; Moreno-Pérez et al. 2017). Therefore, a high vaccination status could not be expected for the examined recruits.

The lack of knowledge of many soldiers of their own vaccination status also indicates that many soldiers are not sufficiently informed of the preventive effect of vaccination or consider this potential protective effect to be of minor importance. The reasons for a negative stance towards vaccination stated in the study by Balicer et al. (2007) could also be due to a lack of information on vaccination.

In summary, all examined soldiers showed large vaccination gaps so that great effort is needed in the relevant armies to establish full protection. This reduces the basic operational readiness of the armed forces and threatens the health of the individual soldiers in case of short-notice operations, especially disaster relief.

## Footnotes

1 Share of the vaccinated population in the total population.

2 For better readability, only the masculine form is used in the following text, this always refers to female members of the armed forces.
